# Validation of the Italian version of the Myasthenia Gravis Impairment Index (MGII)

**DOI:** 10.1007/s10072-021-05585-5

**Published:** 2021-09-09

**Authors:** Francesca Pasqualin, Carolina Barnett, Silvia Vittoria Guidoni, Elisa Albertini, Mario Ermani, Domenico Marco Bonifati

**Affiliations:** 1 Unit of Neurology, Ospedale Regionale Ca’ Foncello, 31100 Treviso, Italy; 2 Division of Neurology, Department of Medicine, University Health Network, Toronto, Canada; 3 Department of Neuroscience, University of Padova, 35128 Padua, Italy

**Keywords:** MGII, Myasthenia gravis, Impairment index, Validation, Italian

## Abstract

**Objective:**

To validate the Italian version of the Myasthenia Gravis Impairment Index (MGII).

**Introduction:**

MGII is a recent promising measure developed for MG patient evaluation. It includes a clinical severity evaluation and a patient-reported questionnaire. It has been developed in English and has demonstrated feasibility, reliability, and construct validity. Recently, its Dutch translation has been validated.

**Methods:**

MGII was translated to Italian with a multi-step forward process. We assessed correlations with the following scores: Istituto Nazionale Carlo Besta score for Myasthenia Gravis (INCB-MG), the MG Activities of Daily Living (MG-ADL), the Myasthenia Gravis Composite (MGC), the Quality of Life 15 for Myasthenia Gravis (QOL15-MG), and the Myasthenia Gravis Disability (MGDIS). We also assessed differences in MGII scores by disease severity with the ANOVA Kruskal–Wallis test.

**Results:**

One hundred forty-one patients were enrolled. The mean MGII total score was 13.3 ± 11.9 (range 0–49), with a mean ocular subscore of 3.7 ± 4.7 and a mean MGII generalized subscore of 9.6 ± 9.0. As expected, the MGII had a good correlation with the other severity scores. The MGII had a lower floor effect (3.5%) than the other measures. Twenty-five patients were assessed in follow-up; as expected, the MGII change scores had moderate correlations with change in other MG severity measures and lower correlations with quality of life measures.

**Conclusions:**

The MGII score was cross-culturally validated in an Italian cohort of MG patients. We confirmed its lower floor effect and the correlations with other MG measures including INCB-MG that was not evaluated in previous studies.

## Introduction

Myasthenia gravis (MG) is an autoimmune disorder due to antibodies against post-synaptic membrane proteins. Impairment in neuromuscular transmission results in fatigue and muscle weakness. Typically, symptoms fluctuate during the day or from day to day, challenging clinical evaluation of patients. This is why many MG measures include the patients’ report of their symptoms (e.g., the ADL (Activity of Daily Living); the MGC (Myasthenia Gravis Composite)), or other measures of fatigability, e.g., INCB-MG [1, 2].

The Myasthenia Gravis Impairment Index (MGII) is a novel measure of myasthenia gravis (MG) severity, with demonstrated feasibility, reliability, and construct validity [3, 4].

The scale has 22 patient-reported items referring to a 2-week recall time and 6 examination items that reflect severity and fatigability of ocular, bulbar, and limb/generalized impairments. The MGII has been formally validated in English and Dutch and is under validation in Spanish and German. However, no formal validation in Italian exists; our aim is to validate the MGII in Italian.

## Methods

Patients were prospectively and consecutively enrolled on a voluntary basis at the myasthenia outpatient clinic of Unit of Neurology, Ca’ Foncello Hospital Treviso, between June and November 2020 during their regular clinical follow-up visit. Patients older than 18 years were included. Diagnosis of myasthenia gravis was confirmed when at least two of the following criteria were present: clinical features typical of MG; serum antibodies against neuromuscular junction proteins; abnormal neurophysiological tests (repetitive nerve stimulation or single-fiber electromyography); positive edrophonium/tensilon test.

The study was approved by the local ethics committee, and written consent from all patients was obtained. The study was planned and carried out in accord with the Helsinki declaration of 1975.

The MGII is a novel measure of MG impairment with demonstrated feasibility, reliability, and construct validity [3, 4]. The scale has 22 patient-reported items referring to a 2-week recall time and 6 examination items. The 6 examination items explore diplopia, ptosis, facialis inferior strength, and arm, leg, and neck endurance. The 22 patient-reported items explore severity and daily and activity-related fatigability of the following symptoms: double vision and droopy eyelids (3 each), swallowing (1), chewing (2), voice and speech articulation (3 each), breathing (1), overall physical tiredness (1), arm weakness (2), leg weakness (2), neck weakness (1). The total scores range between 0 and 84, but it can also be divided into an ocular (0–23) and a generalized (0–61) score; higher scores indicate greater disease severity.

Besta Neurological Institute Rating Scale for MG (INCB-MG) and MG-Composite (MGC) was used to assess patients’ clinical status. The first one is a rating scale developed for MG. It assesses muscle strength and fatigability in 4 muscular districts: ocular, generalized, bulbar, and respiratory. The MGC is a 10-item score for evaluating MG signs and symptoms [5].

MG duration, autoantibody profile, and ongoing medical therapy were derived from clinical records.

In addition to the MGII, the ADL, the MG-QOL15, and the MGDIS were also administered.

The QOL15 is the simplified version of the 60 questions MG-QOL. The 15 questions investigate the impact of the disease on patients’ quality of life. This questionnaire is self-reported by the patient referring to the last 4 weeks. The patient is asked to answer each question on a Likert scale from 0 to 4 (0 = not at all, 4 = totally true). The final score is the sum of each answer and a higher score corresponds to a worse quality of life [6].

The MGDIS is another self-reported questionnaire composed of 20 questions referring to the last month. Each question is scored between 1 and 5 (1 = not at all, 5 = totally true). Like the previous score, a higher sum indicates a more severe disease [7, 8].

MG-ADL is an eight-question measure of MG-related symptoms and activities of daily life. The form is filled by the MD based on the patient’s answers to the questions [9].

### Translation of MGII

In the first phase, the MGII was translated from English into Italian. The translation was carried out with a multi-step forward method: three experimenters (FP, SVG, DMB) independently translated the text of the questionnaire from English into Italian and subsequently discussed the drafting to correct version in a collegial way. This was then subjected to revision by an English mother tongue translator for its verification and correction. Ten patients with disease duration over 5 years were then enrolled for a preliminary analysis of the comprehensibility and clarity of the questions.

The translation was literal for all the items except ITEM 21: leg weakness. The original items refer to weakness after walking a number of blocks. In Italy, “blocks” are not commonly used as distance measure so they have been converted to meters.

### Population

We classified MG patients in the following subgroups: ocular (symptoms strictly ocular for at least two years from onset), early-onset MG-EOMG (generalized anti-acetylcholine positive with age at onset < 50 years), late-onset MG-LOMG (generalized anti-acetylcholine positive with age at onset ≥ 50 years), anti-MuSK MG, double seronegative and thymoma-associated MG.

### Sample size

To calculate the sample size we used the minimal correlation expected in the construct validity studies. For a minimal correlation of *r* = 0.4, with alpha = 0.05 and 90% power, a minimum of 62 patients are needed. COSMIN recommends a minimum of 100 patients. We recruited more (131) to get a better understanding of the performance across the disease spectrum.

### Interrater and test-restest reliability

We tested interrater reliability (IRR) for the examination items on the same day, with a rest period of 30 to 60 min between the 2 raters who were blinded to each other’s scores. IRR was tested with the weighted kappas for the examination items.

Patients returning to a second visit were asked whether they felt better, worse, or unchanged, and only stable patients were included in the test–retest calculations.

Test–retest reliability was tested with the ICC for total score and subscales using a random-effects model (ICC 2, 1)0.23 ICC values > 0.8 are recommended for group and > 0.9 for individual use.

There is no universal consensus on the interpretation of kappa, but usually, values between 0.6 and 0.8 are considered substantial and 0.8 excellent agreement. Finally, we calculated the standard error of measurement.

### Data analysis

Continuous variables were reported as means ± SD (standard deviation), categorical as frequencies or percentages. Data was analyzed with Med Calc and Stata softwares. To reproduce earlier reported construct validity findings of the MGII, we analyzed correlations between MGII and other outcome measures, and we expected similar correlation coefficients (Spearman) than in the original validation study [3]. We assessed differences in MGII between patients with different disease severity indices, measured by the MGFA class, using the ANOVA Kruskal–Wallis test. We expected higher MGII scores with increasing MGFA class. We also compared mean MGII generalized subscores between patients with pure ocular and generalized disease. We also assessed floor effects (proportion of patients with a score = 0) for all the disease severity measures.

Longitudinal validity was assessed with Spearman rank correlation between different measure changes. Significance was set at *p* < 0.05.

## Results

### Population and MGII

141 patients were included in the study, 74 females, 67 males. The mean age was 61.4 ± 15.0, higher in males than in females (67.7 ± 11.5 vs 55.6 ± 15.5 *p* < 0.00001).

The sample included patients with the following subtypes of MG: 20 with ocular MG (3 females, 17 males), 29 EOMG (24 females, 5 males), 47 LOMG (18 females, 29 males), 15 thymoma-associated MG (10 females, 5 males), 11 anti-MuSK positive (9 females, 2 males), and 19 double seronegative (10 females, 9 males).

The mean age at disease onset was 52.1 ± 19.1 and it was higher in males than in females (60.3 ± 15.9 vs 44.6 ± 18.8 *p* < 0.00001). The mean disease duration was 9.6 ± 11.3. It was higher in females than in males ( 11.6 ± 12.3 vs 7.4 ± 9.7 *p* = 0.014) (Table 1).

**Table 1:** Clinical characteristics of the sample

Type of MG (no. pts)		OCULAR (20)	EOMG (29)	LOMG (47)	THYMOMA (15)	SN (19)	ANTI-MUSK (11)	Total (141)
Sex, female/male		3/17	24/4	18/29	10/5	10/9	9/2	74/67
Average age		71.4 ± 11.0	45.9 ± 13.7	69.6 ± 10.7	61.2 ± 14.6	56.0 ± 11.5	57.8 ± 6.9	61.4 ± 15.0
Average age at onset		67.0 ± 12.7	28.5 ± 9.9	64.4 ± 11.3	46.2 ± 17.0	49.4 ± 14.2	47.1 ± 16.5	52.1 ± 19.1
Disease duration		4.3 ± 5.3	17.4 ± 12.0	5.2 ± 3.8	15.0 ± 13.5	9.2 ± 15.3	10.7 ± 14.3	9.6 ± 11.3
MGFA class at enrolment	CSR/PR	7	3	12	3	2	1	28
	MM	12	13	19	5	9	5	63
	MGFA I	1	1	2	2	3		9
	MGFA II		5	8	1	1	1	16
	MGFA III		2	2	3	1	2	10
	MGFA IV		5	4	1	3	2	15

Abbreviations: *CSR/PR* complete stable remission/pharmacological remission, *EOMG* early-onset myasthenia gravis, *LOMG* late-onset myasthenia gravis, *MM* minimal manifestation, *MGFA* Myasthenia Gravis Foundation of America, *MUSK* muscle-specific tyrosine kinase, *SN* double seronegative

The mean MGII total score was 13.3 ± 11.9 (range 0–49), with a mean ocular subscore of 3.7 ± 4.7 and mean MGII generalized subscore of 9.6 ± 9.0.

**Fig. 1:**
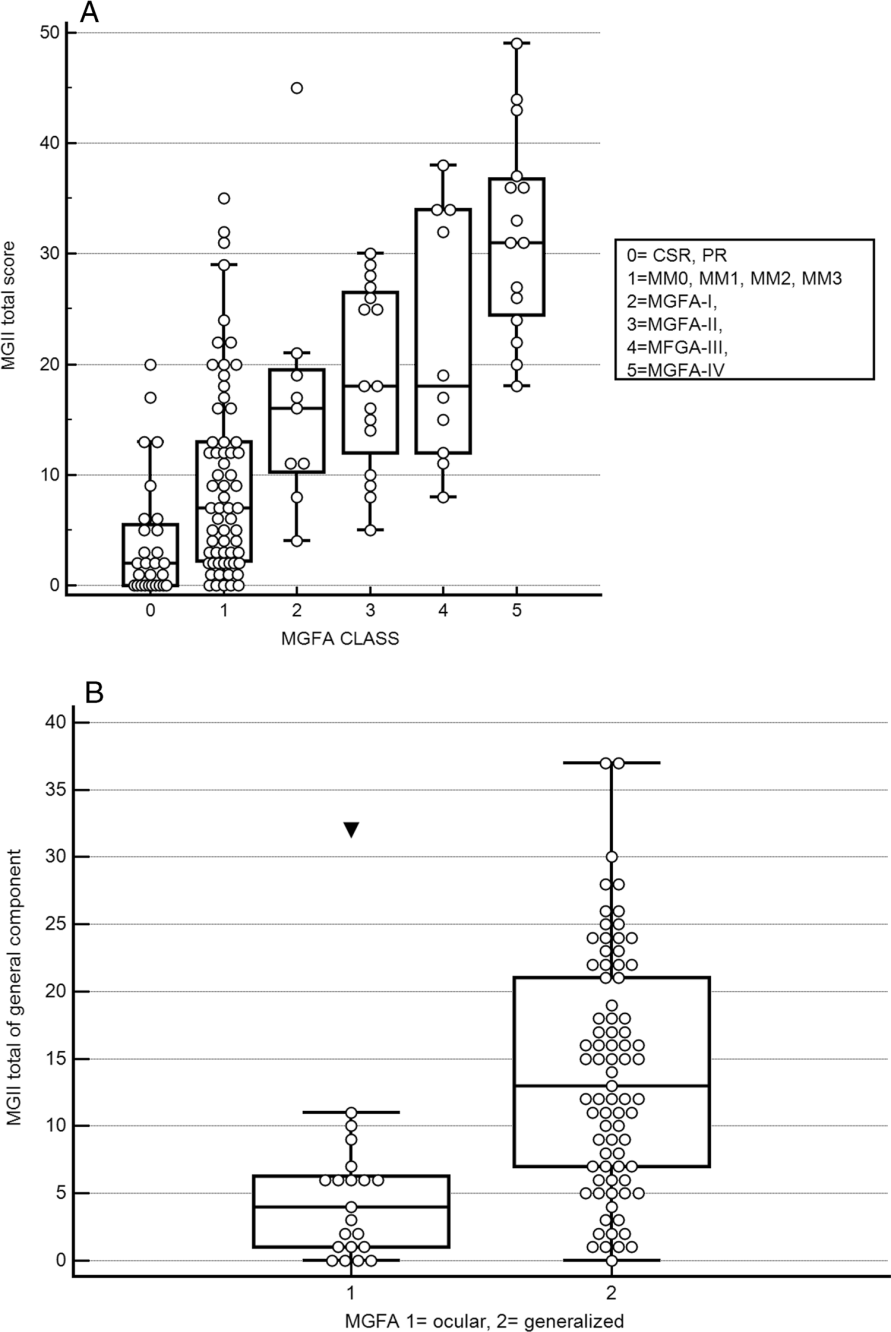
**A** Total scores according to different MGFA classes. Patients in remission had very low total scores (mean 3.96, median 2.0), and scores increased progressively with higher MGFA class (*p* < 0.000001). **B** Generalized subscores in patients with the ocular and generalized disease. As expected, the MGII total score was lower in patients with ocular compared to generalized disease (11.5 ± 10.1 and 18.7 ± 11.8 *p* = 0.008) and ocular patients had minimal scores in the generalized subscore (mean 5.4 ± 7.0)

As in the original validation work for MGII [3] patients in remission had very low total scores (mean 3.96, median 2.0), and scores increased progressively with higher MGFA class (*p* < 0.000001, Fig. 1A).

As expected, the MGII total score was lower in patients with ocular compared to generalized disease (11.5 ± 10.1 and 18.7 ± 11.8 *p* = 0.008) and ocular patients had minimal scores in the generalized subscore (mean 5.4 ± 7.0; Fig. 1B).

### Reliability

Twenty-four patients were assessed for interrater reliability. All items had weighted kappa values between 0.538 and 0.843. Exceptions were the examination item for lower face strength (weighted kappa 0.467 with high agreement). This is due to the “kappa paradox,” when all items had 0 and 1 scores and very high agreement, a case where kappa values are meaningless. The sum of the examination items had good interrater reliability with ICC of 0.747 (95% CI 0.60–0.89). These results are comparable to the ones of the original MGII development study.

Forty-eight patients returning for visit 2 were assessed for test–retest reliability. Of the returning patients, 27 were unchanged from baseline and were included in test–retest reliability. Test–retest reliability was excellent with an ICC of 0.93 (95% CI 0.86–0.97) for the total score and 0.91 (95% CI 0.81–0.96) for the PR items.

### MGII correlation with other scores

Table 2 shows the correlations between MGII and the other measures. MGII had a lower floor effect (3.5%) compared to the MG-ADL (17.7%), MGC (10.6%), QOL15 (9.92%), and MGDIS (4.25%); it was the same as the INCB scale (3.5%).

**Table 2:** Correlations with MGII

Comparison measure	Spearman *R*	95% confidence interval for rho	*p* value
ADL	0.787	0.715 to 0.843	*p* < 0.0001
MGC	0.748	0.665 to 0.813	*p* < 0.0001
QOL15	0.726	0.637 to 0.796	*p* < 0.0001
MGDIS	0.764	0.685 to 0.826	*p* < 0.0001
INCB-MG	0.660	0.555 to 0.745	*p* < 0.0001

Abbreviations: *ADL* Activity of Daily Living, *INCB-MG* Istituto Nazionale Carlo Besta for Myasthenia Gravis, *MGC* myasthenia gravis composite, *MGDIS* Myasthenia Gravis Disability, *QOL15* Quality of Life 15

Twenty-five patients participated in 2 consecutive follow-ups with the administration of all the scores. We investigated if there was a correlation between MGII change and other measures change. As expected, MGII change scores had a moderate correlation (0.426 to 0.600) with changes in other severity measures (ADL, INCB, MGC) and a low correlation with quality of life and disability scores (0.291 with QOL15 and 0.3949 with MGDIS).

## Discussion

The MGII scale was cross-culturally validated into Italian. The translation was literal for all the items except ITEM 21: leg weakness, which was converted from blocks to meters to be more culturally appropriated. This was also made in the Dutch cohort in the corresponding validation process [10].

We validated the Italian version of the MGII in a cohort of 141 Italian MG patients. It has shown excellent validity and reliability as well as the original English version. We found high correlations with other outcome measures, within the hypothesized ranges of original validation [3]. In our study, the correlations with the MG-ADL and MGC were slightly lower than the original study (0.787 and 0.748 vs 0.91 and 0.81 respectively). This can be attributed to a major prevalence of remission (complete or pharmacological) and minimal manifestation status in our sample. This can influence the correlation due to a higher floor effect of the MG-ADL and MGC scores. As expected, patients in remission had very low scores and scores increased with progressively higher MGFA class, which is further evidence of construct validity. We also found low scores in the generalized component in patients with pure ocular MG, in keeping with the original validation. In addition, we replicated previous findings of lower floor effect than the MGC and MG-ADL, and similar to the INCB which was not previously studied.

Despite the variability of muscle weakness in MG, change in MGII in two consecutive visits showed a moderate correlation (0.426 to 0.600) with changes in other severity measures (ADL, INCB, MGC). These values are lower than previously reported but this can be due to the larger sample, variable timing of the second follow-up, and variable interventions. As described in previous MGII studies, the change in MGII correlated better with activity of daily life (ADL) and clinical measures (MGC, INCB) than with change in quality of life and disability scores [4].

Limitations of this study are the single-center recruitment, the variable timing of the second follow-up, and a large number of patients in remission or with minimal manifestations. Another limitation is the lack of correlation with QMG but even if it is largely used in clinical trials, it is less used in clinical practice due to the necessity of a dynamometer. The task force on MG study design of the Medical Scientific Advisory Board of Myasthenia Gravis Foundation in 2012 recommended MGC over QMG because it is “weighted for clinical significance and incorporates patient-reported outcomes.” The strengths are the large sample evaluated at different stages of the disease, and the correlation with INCB which has not been evaluated in previous works.

The MGII was cross-culturally validated in Italian, with evidence of construct validity, strong reliability, and low floor effect in an Italian population of Myasthenia Gravis patients with different cultural backgrounds. We recommend including the MGII in future myasthenia clinical trials.

## Data Availability

The data that support the findings of this study are available from the corresponding author upon reasonable request.

## Abbreviations

EOMG: Early-onset myasthenia gravis
INCB-MG: Istituto Nazionale Carlo Besta Score for Myasthenia Gravis
LOMG: Late-onset myasthenia gravis
QOL15-MG: Quality of Life 15 for Myasthenia Gravis
MG: Myasthenia gravis
MG-ADL: MG Activities of Daily Living
MGC: Myasthenia Gravis Composite
MGDIS: Myasthenia Gravis Disability
MGFA: Myasthenia Gravis Foundation of America
MGII: Myasthenia Gravis Impairment Index
Musk: Muscle-specific tyrosine kinase
SN: Double seronegative
